# Zinc Deficiency and Post-acute Outcomes in Patients With COVID-19: A Six-Month Retrospective Cohort Analysis of 3,726 Patients

**DOI:** 10.7759/cureus.71609

**Published:** 2024-10-16

**Authors:** Lun-Wu Hung, Mei-Yuan Liu, Tsung Yu, Kuo-Chuan Hung, Ya-Wen Tsai, Chih-Cheng Lai, Jheng-Yan Wu

**Affiliations:** 1 Division of Cardiovascular Surgery, Department of Surgery, Chi Mei Medical Center, Tainan, TWN; 2 Department of Nutrition, Chi Mei Medical Center, Tainan, TWN; 3 Department of Public Health, College of Medicine, National Cheng Kung University, Tainan, TWN; 4 Department of Anaesthesiology, Chi Mei Medical Center, Tainan, TWN; 5 Division of Preventive Medicine, Chi Mei Medical Center, Tainan, TWN; 6 Department of Intensive Care Medicine, Chi Mei Medical Center, Tainan, TWN

**Keywords:** covid-19, long covid-19, mortality, post-acute covid-19, zinc deficiency

## Abstract

Background

Previous studies have suggested that zinc deficiency (ZD) may increase the risk of short-term mortality in patients with coronavirus disease 2019 (COVID-19). However, the relationship between zinc status and post-acute COVID-19 outcomes remains unclear. This study aimed to determine the association between ZD and long-term outcomes in patients with COVID-19.

Methodology

We conducted a retrospective cohort study using the TriNetX database, including patients aged 18 years or older diagnosed with COVID-19 between January 1, 2022, and July 31, 2023. Patients had documented serum or plasma zinc levels within three months before COVID-19 diagnosis and were not deceased or hospitalized in the first month of infection. They were categorized into ZD (zinc levels <70 μg/dL) and control (zinc levels ≥70 μg/dL) groups. After 1:1 propensity score matching for demographic and clinical variables, outcomes were assessed from 30 to 180 days post-diagnosis, including all-cause hospitalization, all-cause mortality, and four subphenotypes of post-acute COVID-19. Hazard ratios (HRs) with 95% confidence intervals (CIs) were calculated.

Results

After matching, each group included 1,863 patients with balanced baseline characteristics. The ZD group had a higher incidence of all-cause hospitalization (25.3% vs. 20.3%; HR = 1.314; 95% CI = 1.148-1.505; p < 0.001) and all-cause mortality (3.8% vs. 2.2%; HR = 1.735; 95% CI = 1.180-2.551; p = 0.045) compared to the control group during the follow-up period. Among the four subphenotypes, only the cardiac and renal subphenotype showed a significantly higher risk in the ZD group (HR = 1.099; 95% CI = 1.002-1.205; p = 0.004).

Conclusions

ZD is associated with increased risks of long-term hospitalization, mortality, and increased risk in COVID-19 patients with cardiac and renal comorbidities. Monitoring and managing zinc levels may be important for improving long-term outcomes. Further research is warranted to explore the potential benefits of zinc supplementation in COVID-19 patients with ZD.

## Introduction

Zinc deficiency (ZD) has been increasingly recognized as a factor that may influence the clinical outcomes of patients with coronavirus disease 2019 (COVID-19). Several studies have assessed the association between ZD and short-term clinical outcomes in COVID-19 patients [1-3]. Wu et al. demonstrated that ZD might increase the risk of 30-day mortality in COVID-19 patients [1]. Additionally, previous systematic reviews and meta-analyses have shown that zinc supplements may have a borderline or small effect on reducing mortality rates [2,3]. These findings suggest that zinc plays a crucial role in the immune response to severe acute respiratory syndrome coronavirus-2 (SARS-CoV-2) infection.

Despite the evidence linking ZD to short-term outcomes, the relationship between zinc status and post-acute COVID-19 outcomes remains unclear. The post-acute sequelae of COVID-19, often referred to as “long COVID,” encompasses a variety of symptoms affecting multiple organ systems, which can persist for months after the initial infection. Understanding factors that contribute to these long-term outcomes is essential for improving patient care and developing targeted interventions.

Given this knowledge gap, this study aims to explore how ZD affects hospitalization and mortality rates in COVID-19 patients over six months.

## Materials and methods

Database

We conducted the study using the TriNetX database, which provides real-time electronic medical records (i.e., data regarding diagnoses, procedures, medications, laboratory test values, and genomic information) of approximately 129 million patients from 108 healthcare organizations [4]. As a federated network, TriNetX has been awarded an exemption by the Western Institutional Review Board. This is due to its operation model, which solely involves handling aggregated and deidentified statistical data, ensuring it remains without access to any protected health information and is not engaged in any specific study-related activities during the analysis of retrospective data. This study was approved by the Institutional Review Board of the Chi Mei Medical Center (approval number: 11302-E01).

Patient selection

We enrolled patients who (a) were aged ≥18 years; (b) were diagnosed with COVID-19 (an International Classification of Diseases, Tenth Revision, Clinical Modification (ICD-10-CM) code with U07.1), or had a recorded positive polymerase chain reaction test for COVID-19 (a laboratory test with TNX: LAB:9088) as previously described [5-7]; (c) had a laboratory test of zinc levels in serum or plasma (a laboratory test with TNX: LAB:5763-8) within three months before COVID-19; (d) were not deceased or hospitalized in the first month of COVID-19; and (e) had at least two visits to HCOs between January 1, 2022, and July 31, 2023. The enrolled patients were categorized into two groups based on their zinc levels. The ZD group had zinc levels <70 μg/dL, whereas the control group had zinc levels >70 μg/dL [8]. The two groups were assembled using 1:1 propensity score matching considering age, race, gender, comorbidities, and adverse socioeconomic determinants of health.

The index date was defined as the first date of COVID-19 diagnosis, and the patients were followed up from 30 days after the index date until the incidence of outcomes, or up to 180 days from the index date. The outcomes included the risk of post-acute COVID-19, which encompassed (a) all-cause hospitalization; (b) all-cause mortality; and (c) the following four subphenotypes of post-acute COVID-19 [9]: subphenotype 1 (cardiac and renal) identified by ICD-10-CM code with E87, D63, D64, R00, N17, N19, I50, I49, R11, R10, and R53; subphenotype 2 (respiratory, sleep and anxiety) identified by ICD-10-CM code with R51, G47, J40-J4A, J45, R06, R07, R68, R00, R10, J45, N17, N19, R40.4, R41, R48, G93.4, G31.84, F02, F03, F05, F06, G30, and E87; subphenotype 3 (musculoskeletal and nervous) identified by ICD-10-CM code with M45-M49, M65-M67, M35, M15-M19, M79.1, G47, G89-G99, T85.84, T85.840S, T85.840A, T85.840D, R40.4, R41, R48, G93.4, G31.84, F02, F03, F05, F06, G30, R51, R53, R20.0, R21, and L00-L08; and subphenotype 4 (digestive and respiratory) identified by ICD-10-CM code with K20-K31, R51, G47, J40-J4A, J45, R07, R06, R68, D63, D64, R10, R53, and R11. The hazard ratio (HR) with 95% confidence intervals (CIs) of the outcomes was calculated for the ZD group versus the control group.

Statistical analysis

All statistical analyses were conducted using the built-in function of the TriNetX network.

## Results

**Figure 1:**
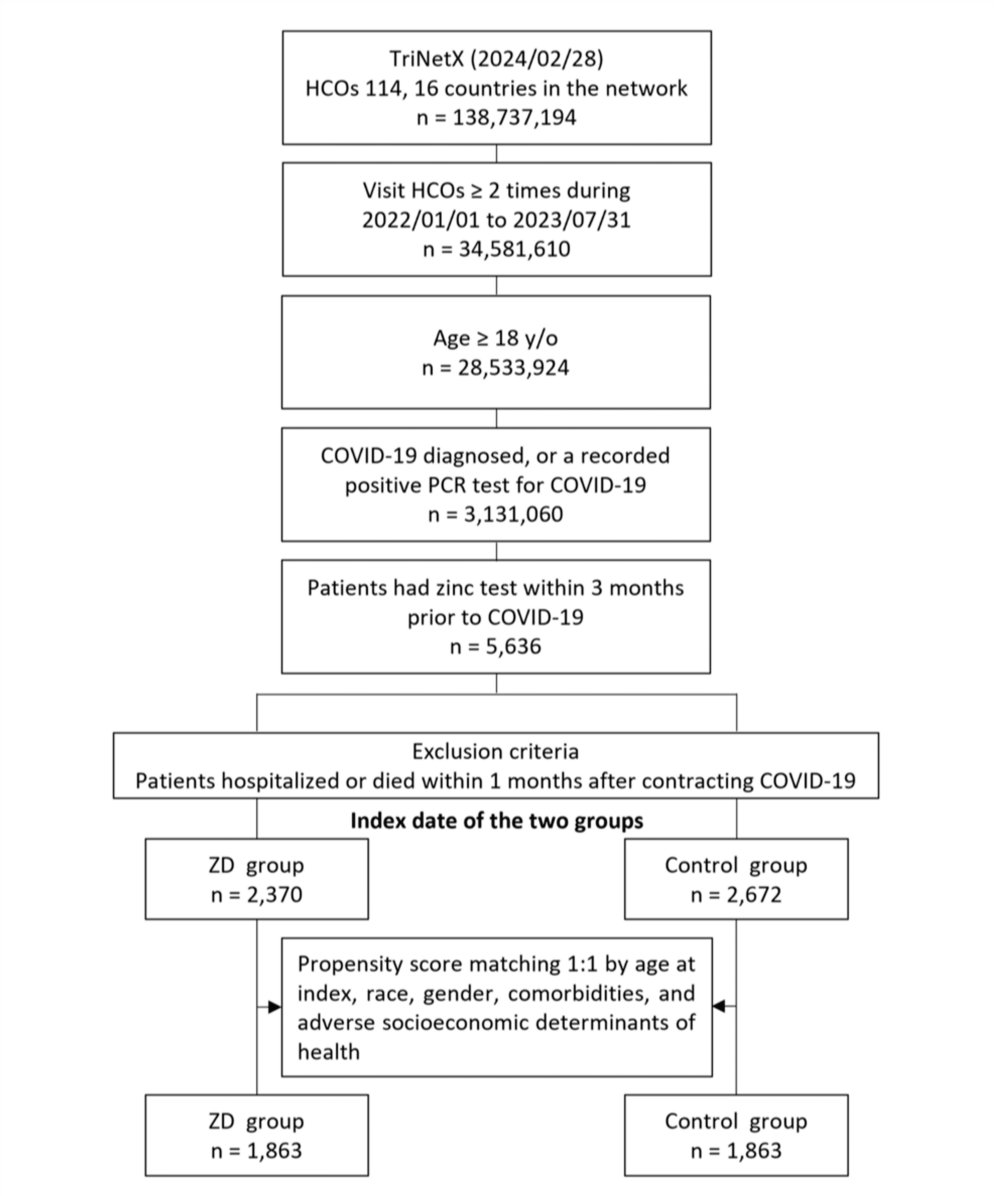
Flowchart of patient selection and cohort construction. ZD: zinc deficiency

Initially, 5,636 patients who met the inclusion criteria were included, of these, 2,370 were classified into the ZD group, and the remaining 2,672 were assigned to the control group (Figure 1).

The baseline characteristics of the study population before and after propensity score matching are presented in Table 1. Before matching, significant differences were observed between the ZD group (n = 2,370) and the control group (n = 2,672) in several demographic and clinical variables. The mean age was higher in the ZD group compared to the control group (55.0 ± 18.0 years vs. 50.5 ± 16.8 years; p < 0.001). A lower proportion of female patients was noted in the ZD group (62.8%) compared to the control group (68.3%; p < 0.001). Racial distribution differed significantly, with the ZD group having a lower percentage of White patients (61.6% vs. 66.8%; p < 0.001) and a higher percentage of Black or African American patients (17.7% vs. 12.8%; p < 0.001). Regarding social determinants of health, problems related to housing and economic circumstances were more prevalent in the ZD group than in the control group (5.6% vs. 4.3%; p = 0.032). In terms of comorbidities, the ZD group exhibited higher rates of malnutrition (27.4% vs. 13.6%; p < 0.001), alcohol-related disorders (12.7% vs. 7.5%; p < 0.001), and nicotine dependence (19.3% vs. 14.4%; p < 0.001). Cardiovascular conditions were also more common in the ZD group, including essential hypertension (59.5% vs. 50.9%; p < 0.001), heart failure (23.5% vs. 12.3%; p < 0.001), cerebrovascular diseases (21.2% vs. 13.2%; p < 0.001), atrial fibrillation and flutter (15.6% vs. 9.8%; p < 0.001), and ischemic heart diseases (28.1% vs. 18.2%; p < 0.001). Additionally, the ZD group had higher prevalences of diabetes mellitus (36.5% vs. 30.6%; p < 0.001), chronic kidney disease (26.2% vs. 14.4%; p < 0.001), and end-stage renal disease (7.0% vs. 3.6%; p < 0.001).

**Table 1:** Baseline characteristics of the study population before and after propensity score matching. Continuous variables were compared using an independent t-test, and categorical variables were compared using the chi-square test. An alpha level of 0.05 was selected for statistical significance. ZD: zinc deficiency

Variables	Before matching			After matching		
	ZD group (n = 2,370)	Control group (n = 2,672)	P-value	ZD group (n = 1,863)	Control group (n = 1,863)	P-value
Age at index, years						
Mean ± SD	55.0 ± 18.0	50.5 ± 16.8	<0.001	52.8 ± 17.9	52.2 ± 17.5	0.352
Gender, n (%)						
Female	1,489 (62.8)	1,826 (68.3)	<0.001	1,240 (66.6)	1,239 (66.5)	0.972
Male	795 (33.5)	685 (25.6)	<0.001	550 (29.5)	543 (29.1)	0.801
Race, n (%)						
White	1,459 (61.6)	1,784 (66.8)	<0.001	1,201 (64.5)	1,196 (64.2)	0.864
Black or African American	419 (17.7)	342 (12.8)	<0.001	291 (15.6)	292 (15.7)	0.964
Asian	65 (2.7)	51 (1.9)	0.049	43 (2.3)	42 (2.3)	0.913
Unknown race	266 (11.2)	328 (12.3)	0.248	202 (10.8)	208 (11.2)	0.753
Social determinants of health associated with adverse outcomes, n (%)						
Problems related to housing and economic circumstances	132 (5.6)	114 (4.3)	0.032	89 (4.8)	96 (5.2)	0.598
Problems related to employment and unemployment	49 (2.1)	72 (2.7)	0.146	44 (2.4)	43 (2.3)	0.914
Problems related to education and literacy	13 (0.5)	13 (0.5)	0.759	10 (0.5)	10 (0.5)	1.000
Occupational exposure to risk factors	10 (0.4)	14 (0.5)	0.599	10 (0.5)	10 (0.5)	1.000
Comorbidities, n (%)						
Overweight and obesity	1,075 (45.4)	1,352 (50.6)	<0.001	884 (47.5)	893 (47.9)	0.768
Malnutrition	649 (27.4)	363 (13.6)	<0.001	344 (18.5)	339 (18.2)	0.832
Alcohol-related disorders	302 (12.7)	201 (7.5)	<0.001	176 (9.4)	167 (9)	0.610
Nicotine dependence	457 (19.3)	384 (14.4)	<0.001	296 (15.9)	304 (16.3)	0.721
Essential hypertension	1,411 (59.5)	1,360 (50.9)	<0.001	1,014 (54.4)	1,034 (55.5)	0.510
Dyslipidemia	1,089 (45.9)	1,319 (49.4)	0.015	835 (44.8)	856 (45.9)	0.490
Diabetes mellitus	865 (36.5)	817 (30.6)	<0.001	618 (33.2)	632 (33.9)	0.627
Neoplasms	1,049 (44.3)	1,096 (41)	0.020	782 (42)	794 (42.6)	0.691
Chronic lower respiratory diseases	865 (36.5)	866 (32.4)	0.002	638 (34.2)	656 (35.2)	0.536
Diseases of liver	96 (4.1)	28 (1)	<0.001	28 (1.5)	30 (1.6)	0.791
Chronic kidney disease	620 (26.2)	385 (14.4)	<0.001	360 (19.3)	354 (19)	0.803
End-stage renal disease	165 (7)	97 (3.6)	<0.001	91 (4.9)	84 (4.5)	0.588
Heart failure	558 (23.5)	329 (12.3)	<0.001	307 (16.5)	315 (16.9)	0.725
Cerebrovascular diseases	503 (21.2)	352 (13.2)	<0.001	317 (17)	325 (17.4)	0.729
Atrial fibrillation and flutter	369 (15.6)	261 (9.8)	<0.001	232 (12.5)	234 (12.6)	0.921
Ischemic heart diseases	665 (28.1)	486 (18.2)	<0.001	411 (22.1)	426 (22.9)	0.556

After performing 1:1 propensity score matching, each group consisted of 1,863 patients with well-balanced baseline characteristics (Table 1). The mean ages were similar between the ZD group and the control group (52.8 ± 17.9 years vs. 52.2 ± 17.5 years; p = 0.352). The proportion of female patients was nearly identical in both groups (66.6% vs. 66.5%; p = 0.972). Racial distributions were comparable, with no significant differences in the proportions of White patients (64.5% vs. 64.2%; p = 0.864) or Black or African American patients (15.6% vs. 15.7%; p = 0.964). Social determinants of health related to housing and economic circumstances were similar between the ZD and control groups (4.8% vs. 5.2%; p = 0.598). Comorbidities were also balanced after matching. The prevalence of malnutrition was comparable between the ZD and control groups (18.5% vs. 18.2%; p = 0.832), as were rates of alcohol-related disorders (9.4% vs. 9.0%; p = 0.610) and nicotine dependence (15.9% vs. 16.3%; p = 0.721). Cardiovascular conditions showed no significant differences post-matching, including essential hypertension (54.4% vs. 55.5%; p = 0.510), heart failure (16.5% vs. 16.9%; p = 0.725), cerebrovascular diseases (17.0% vs. 17.4%; p = 0.729), atrial fibrillation and flutter (12.5% vs. 12.6%; p = 0.921), and ischemic heart diseases (22.1% vs. 22.9%; p = 0.556). Similarly, the prevalences of diabetes mellitus (33.2% vs. 33.9%; p = 0.627), chronic kidney disease (19.3% vs. 19.0%; p = 0.803), and end-stage renal disease (4.9% vs. 4.5%; p = 0.588) were not significantly different between the two groups.

**Figure 2:**
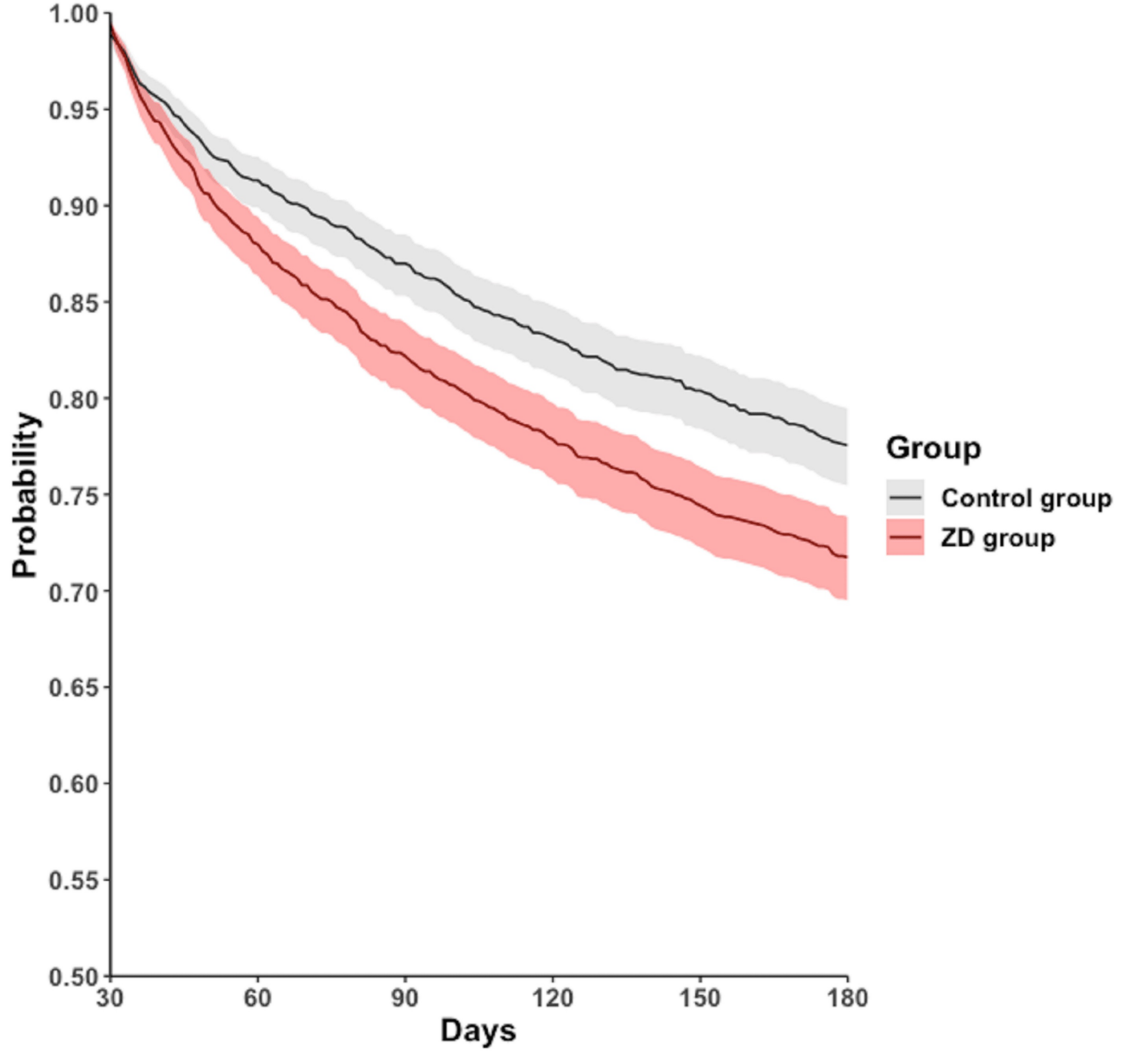
Kaplan-Meier time-to-event free curves for the all‐cause hospitalization. ZD: zinc deficiency

**Figure 3:**
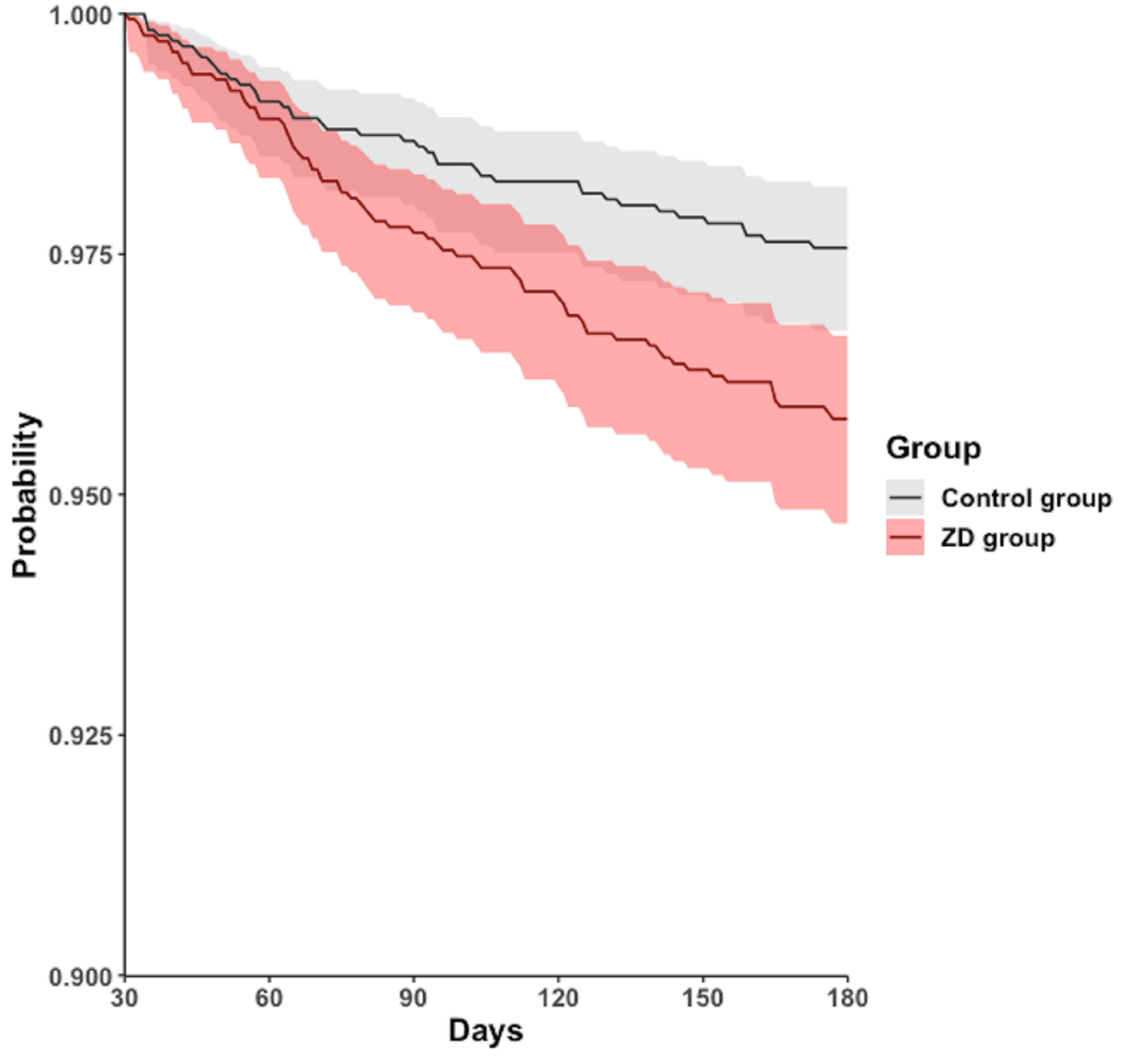
Kaplan-Meier time-to-event free curves for the all-cause mortality. ZD: zinc deficiency

During the follow-up period of 30-180 days, 472 (25.3%) patients in the ZD group and 378 (20.3%) in the control group experienced all-cause hospitalization. A similar trend was observed in all-cause mortality, with 70 (3.8%) patients in the ZD group and 41 (2.2%) in the control group. The Cox regression model and Kaplan-Meier plot showed that, compared with the control group, the ZD group had a higher cumulative curve for time-to-even free probability within 30-180 days, indicating a significantly higher incidence of all-cause hospitalization (HR = 1.314; 95% CI = 1.148-1.505; log-rank test; p < 0.001) (Table 2, Figure 2) and all-cause mortality (HR = 1.735; 95% CI = 1.180-2.551; log-rank test; p = 0.045) (Table 2, Figure 3). However, the difference in the four subphenotypes of post-acute COVID-19 between the groups was inconsistent. Only subphenotype 1 (patients with cardiac and renal comorbidities) showed a statistically significant difference with an HR of 1.099 (95% CI = 1.002-1.205 (Table 2), remaining comparisons were insignificant (subphenotype 2: HR = 0.970; 95% CI = 0.891-1.056; subphenotype 3: HR = 1.003; 95% CI = 0.920-1.094; subphenotype 4: HR = 0.957; 95% CI = 0.882-1.038 (Table 2).

**Table 2:** Hazard ratios of clinical outcomes and subphenotypes of post-acute COVID-19 for the matched zinc deficiency group and control group. A Cox regression model was used to compare the hazard ratio between the two groups. An alpha level of 0.05 was selected for statistical significance. CI: confidence interval; ZD: zinc deficiency

Outcomes	Patients with outcome (n)		Hazard ratio (95% CI)	P-value
	ZD group	Control group		
Clinical outcomes				
All‐cause hospitalization	472	378	1.314 (1.148 to 1.505)	<0.001
All-cause mortality	70	41	1.735 (1.180 to 2.551)	0.045
Subphenotypes of post-acute COVID-19				
Subphenotype 1 (cardiac and renal)	928	887	1.099 (1.002 to 1.205)	0.044
Subphenotype 2 (respiratory, sleep, and anxiety)	1,049	1,082	0.970 (0.891 to 1.506)	0.486
Subphenotype 3 (musculoskeletal and nervous)	1,005	1,029	1.003 (0.920 to 1.094)	0.947
Subphenotype 4 (digestive and respiratory)	1,136	1,170	0.957 (0.882 to 1.038)	0.285

## Discussion

In summary, our retrospective cohort study revealed an association between ZD and adverse long-term clinical outcomes in COVID-19 patients. Several factors could contribute to the observed findings. Several factors may contribute to the observed association between ZD and poorer outcomes. Zinc is an essential trace element crucial for maintaining immune function, supporting antioxidant defenses, and regulating inflammatory responses [10]. It plays a vital role in both innate and adaptive immunity, influencing the function of neutrophils, natural killer cells, T lymphocytes, and B lymphocytes. ZD can impair these immune functions, leading to increased susceptibility to infections and a diminished ability to combat viral pathogens such as SARS-CoV-2. In the context of COVID-19, adequate zinc levels may help modulate the immune response, potentially reducing the severity of the disease and preventing the progression to severe complications. The increased risk of cardiac and renal complications observed in ZD patients may be attributed to zinc’s role in cellular metabolism and oxidative stress reduction in these organ systems. The physiological and pathological mechanisms regarding ZD have been associated with impaired endothelial function, increased arterial stiffness, and myocardial dysfunction, all of which can contribute to cardiovascular complications. Similarly, zinc plays a role in protecting against renal injury by reducing inflammation and oxidative stress in the kidneys. In ZD patients, the lack of these protective mechanisms may explain the increased risk of renal complications observed in our study.

The clinical implications of our study are significant. Monitoring zinc levels in patients diagnosed with COVID-19 could serve as an important prognostic tool. Identifying ZD early allows for timely nutritional interventions, such as zinc supplementation, which may improve patient outcomes. Healthcare providers should consider integrating routine nutritional assessments into the management plans for COVID-19 patients, particularly for those with risk factors for micronutrient deficiencies. However, the specific impact of zinc supplementation on long-term COVID-19 outcomes requires further investigation. Randomized controlled trials are needed to determine the efficacy and safety of zinc supplementation as an adjunct therapy for COVID-19 patients with ZD. Future research should explore several key aspects to optimize the potential benefits of zinc supplementation. One important direction is to determine the optimal dosage of zinc supplementation. Identifying a dosage that maximizes therapeutic benefits while minimizing the risk of toxicity is essential. Such research should consider patient-specific factors such as age, comorbidities, and baseline zinc levels to tailor treatment plans for different populations. Another critical area for investigation is the timing of zinc supplementation. The therapeutic window, whether zinc should be initiated during the acute phase of COVID-19, immediately post-diagnosis, or preemptively in high-risk populations, remains unclear. Understanding when zinc supplementation would have the greatest impact could improve patient outcomes and reduce long-term complications. Additionally, future randomized controlled trials should be designed to stratify patients based on their baseline zinc levels and the severity of their COVID-19 infection. Long-term follow-up will be crucial to evaluate the impact of zinc supplementation on specific post-acute complications, particularly cardiac and renal outcomes. By addressing these important areas, future research can provide more comprehensive insights into the role of zinc supplementation in improving the clinical outcomes of COVID-19 patients with ZD.

This study has some limitations. First, due to the limitations of the TriNetX database, we were unable to directly obtain or categorize patients based on COVID-19 severity, as specific clinical indicators such as oxygen supplementation, intensive care unit admission, or other markers of disease severity were not uniformly available in the dataset. Second, although propensity score matching was utilized to balance baseline characteristics and reduce confounding, residual confounders may still exist. Third, important variables such as the severity of COVID-19 at presentation, viral variants, vaccination status, and specific treatments received were not accounted for in our analysis due to data limitations. These factors could influence both zinc levels and clinical outcomes. Additionally, the use of electronic health records from the TriNetX database may introduce variability in data quality and completeness. Laboratory measurements of zinc levels might differ between institutions, and the timing of zinc assessment relative to COVID-19 diagnosis could affect the results. Our study focused on all-cause hospitalization and mortality, which may include events not directly related to COVID-19 or its sequelae. Finally, the observational nature of this study precludes the establishment of a causal relationship between ZD and adverse outcomes. While associations were identified, causality cannot be inferred without further experimental studies.

## Conclusions

Our study suggests that zinc deficiency is associated with increased risks of long-term hospitalization (HR = 1.314; 95% CI = 1.148-1.505; p < 0.001), mortality (HR = 1.735; 95% CI = 1.180-2.551; p = 0.045), and cardiac and renal complications in patients with COVID-19 (HR = 1.099; 95% CI = 1.002-1.205; p = 0.044). These findings underscore the importance of considering zinc status in the management of COVID-19. Routine monitoring of zinc levels and addressing deficiencies through dietary counseling or supplementation may represent a valuable strategy to improve long-term outcomes. Further research, including prospective studies and clinical trials, is warranted to explore the potential benefits of zinc supplementation in this patient population for zinc management in the context of COVID-19.
